# Parental investment in Tibetan populations does not reflect stated cultural norms

**DOI:** 10.1093/beheco/arx134

**Published:** 2017-10-20

**Authors:** Juan Du, Ruth Mace

**Affiliations:** 1 Department of Anthropology, UCL, London, UK; 2 Life Sciences, Lanzhou University, Lanzhou, Gansu Province, PRC

**Keywords:** inheritance system, mismatch, pastoralist, sex-preference, social norms

## Abstract

In this paper, we examined both stated norms of sex preference and actual sex-biases in parental investment in a Tibetan pastoralist society. We collected detailed demographic data on infant mortality, infant feeding, the length of interbirth intervals, and a decision when giving gifts, to examine sex-biased parental investment. Our results indicate a mismatch between self-reported son preference and measures of actual parental investment that favor daughters. We interpret this female-biased parental investment as a possible response to daughters generating more economic resources. However, the stated sex preferences of both sexes reflect cultural norms that appear to have remained unchanged over a long period, which may reflect the importance of male roles in the past. Our behavioral measures of parental investment are those most likely to be in the control of women (such as breastfeeding and interbirth interval), so this mismatch between stated and actual investment may be especially true of women.

## Teaser

The evolutionary processes that generate cultural group norms and individual behavior may differ. We found that stated gender preference is based on the social norm of son preference, whilst real-life biases in parental investment were in favor of daughters. The recent history of the region includes dramatic government policy changes that influenced the behavioral ecology of the system by changing access to resources and inheritance rules. We discuss how male preferences may differ from female preferences; and we discuss how social norms and maternal behavior may be generated by different evolutionary processes.

## INTRODUCTION

Patterns of wealth inheritance and resource transfer associated with marriage are important determinants of the parental investment received by each sex, especially in societies with bridewealth or dowry payment systems (Mace 2010). Behavioral variation can be understood as an adaptation to the local ecology, but several models suggest reliance on social learning can be slow to respond to rapid environmental change (Mesoudi et al. 2015). Cultural norms (in the sense of the shared expectations and rules that guide the behavior of people within social groups), by definition, tend to be considered invariant within the group (Henrich and Boyd 2001; Henrich and Broesch 2011). However, behavior that maximizes individual inclusive fitness may vary between individuals within one population, such as males and females, or older and younger generations (Micheletti et al. 2017). Here we use detailed demographic data from 5 villages in Amdo, China, to examine different measures of parental investment that can reveal sex-biases in parental behavior. We measure sex-biased parental investment through the breast and bottle feeding of infants, the length of birth intervals and investments in others in their family in economic gift games. We show how sex-biased parental investment has changed over a period of considerable upheaval, as government policy has altered who controls resources over the last 50 years (Gates 1993). We found that for most of this period, behavioral measures indicate that daughters have been favored in Amdo Tibet, yet people report their stated preference to be in favor of sons.

Individuals are predicted to prioritize care for descendants in ways that increase their own inclusive fitness (Hamilton 1964). The costs and benefits of parental investment depend on the offsprings’ potential reproductive success based on that investment, be it care (Bereczkei and Dunbar 1997), or inherited resources (Hartung 1976). While female-biased parental investment is recorded in many societies (Alexander RD 1974; Cronk 1989; Holden et al. 2003; He et al. 2016), son-biased parental investment appears to be more common (Williamson 1976), and is certainly the more prevalent norm in China (Murphy et al. 2011). Son-biased investment is commonly found in patrilineal societies where marriage and mating are polygynous and where males generate or control resources (Hartung 1976; Mace 1996; Aitane 2009). In societies where males generally compete for females through wealth ownership, parents of females can often demand a brideprice for their daughters. Where wealth inheritance is a more important determinant of the reproductive success of males than of females, males can become the more costly sex in terms of wealth transfers, whereas daughters can be profitable as a source of brideprice. In monogamous societies, females often compete with each other for a wealthy husband, as all a husband’s resources pass to the offspring of his only wife. Female competition in monogamous societies means that a desirable husband can demand the payment of a dowry by the bride’s parents (Gaulin and Boster 1990); hence females can become the more costly sex to marry off. Among Tibetan groups in China, both monogamy, polygyny, dowry and brideprice are all observed, with wealth transfers being an important factor affecting long-term fitness.

The “Trivers-Willard Hypothesis” (Trivers 1972) predicts that high-quality mothers are more likely to produce or rear sons when there is higher variance in reproductive potential for sons compared to daughters; whereas when mothers are of low socioeconomic status they do better to produce and rear daughters, who show less wealth-related variance in reproductive success. Whilst there is general agreement that this theory predicts sex ratio at birth, its application to parental investment after birth is now thought to be more context dependent (Veller et al. 2016). However, several studies have shown that the birth order of the offspring and socioeconomic status affects son-biased parental investment in different provinces in China (Banister 2004; Aitane 2009; Murphy et al. 2011). In the United States, by contrast, it has been shown that family status has no effect on the sex-biased investment (Keller et al. 2001). Female-biased mortality is expected to increase in China after the implementation of the family planning policy, as restricting births to one or a few children intensifies pressure for the one child to be of the preferred sex, which is usually male (Edlund 1999).

Demographers have noted that sex-biased parental investment is also based on the potential benefits that parents are expecting to get from the offspring when in need (termed “local resource enhancement” by Gowaty and Lennartz [1985]). For example, daughters might be favored in Tibetan societies (Childs et al. 2011) and in other parts of China (Zhan and Montgomery 2003) because they are more likely to provide both emotional and instrumental support for their parents in their later life. Daughters are thought to be favored by mothers in the US Hutterite society because they are more likely to help mothers in babysitting the younger offspring and to be useful in helping with daily household duties (Margulis et al. 1993). Daughters can get more educational investment in Southeast Asian societies, because parents are more likely to get support from their daughters compared to their sons (Degraff and Bilsborrow 1996; Anderson et al. 2003), and daughters offer more help in looking after siblings in Hungarian gypsy populations (Dunbar 2002). Mukogodo parents in Kenya breastfed daughters more and were more likely to take daughters to the clinic because they had higher reproductive success and brought more economic benefits to the family (Cronk 1993). Cronk describes that in India, ancient Germany, ancient Portugal and contemporary North America, there is also female-biased parental investment (Cronk 1991).

Breastfeeding duration can be one measure of maternal investment (Oddy 2001). It is an obligate maternal investment behavior essential for child survival (Dewey 1998; Bezner Kerr et al. 2008). The amount of nutritious support from the mother through breastfeeding can ameliorate the negative effects of poor socioeconomic status on children’s health (Sparks 2011). Early complementary feeding can bring many side effects for child health, sometimes resulting in increased child mortality (Kalanda et al. 2006). Breastfeeding incurs the opportunity cost of time and energy, so breastfeeding mothers are presented with the choice between providing resources for themselves and for their children (WHO 2003; Wilhelm et al. 2008). Birth intervals can be indicative of sex-biased parental investment, if mothers tend to postpone further reproduction after the birth of a particular sex (Mace and Sear 1997; Crognier et al. 2002; Mattison et al. 2015; Mattison et al. 2016; Veller et al. 2016).

Complementary feeding can be dangerous for infants because breast milk is more nutritious and reduces the chance of disease (Martin et al. 2016); but bottle-feeding is very useful to help maintain a mother’s physical condition during a time of high energy requirements (Kramer 2010; Martin et al. 2016). However mothers not only initiate bottlefeeding to less favored offspring, but they sometimes do so to feed big infants that require more nutritional resources (Margulis et al. 1993; Mace and Sear 1997). Decisions about how long to breastfeed and when to initiate complementary feeding influence both the interbirth interval and the survival of children; thus, feeding strategies have a significant influence on reproductive success. Although the decision to have another baby or the degree of investment in offspring comes from both the father and mother, weaning is mainly in the hands of mothers (Mace and Sear 1997). The social and economic status of a woman is an important factor which determines how much energy she is able to invest in production and reproduction (Hare 1999; Fujita et al. 2012; Wander and Mattison 2013).

Before the communist regime in the 1950s, land and livestock were not distributed equally among households in Tibet, leading to wealth inequality. Wealth inequality results in more polygamous marriage and social hierarchy (Levine 2015). In Tibetan history, men played an important role as herders and warriors, especially when the social system was shaped by local warfare with neighboring villages and the raiding of livestock (Huber 2012). Traditional nomadic lifestyles were highly mobile, less buffered from natural disasters (Yeh et al. 2014) and prone to frequent conflicts at the borders of their pastures (Yeh 2003). Male herders were key to safeguarding the family and family livestock, and supplemented the family income with raids on other groups. Traditional Tibetan herders, like many other pastoralist societies (Sieff et al. 1990; Mace 1996), had a preference for sons (Levine 1987).

**Figure 1:**
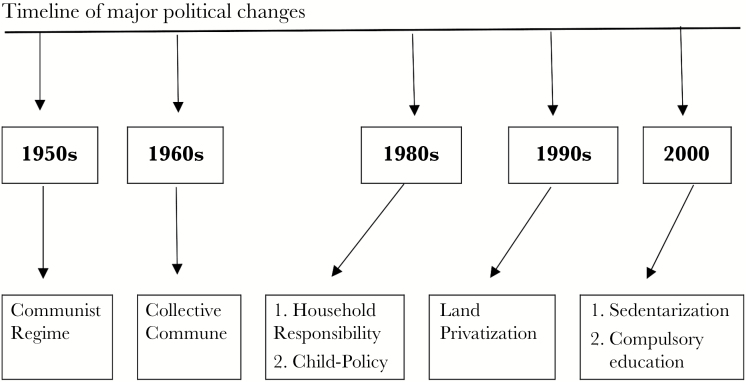
Timeline of the major political changes in the local area from 1950s until 2015 (see SI for details).

The last 70 years in China were characterized by many major political changes, shown below in Figure 1 (see the SI for details). These have influenced how resources are owned and inherited across generations, which has in turn influenced the roles of male and female Tibetan herders. Major changes arose first from the general changes brought by the incoming communist regime when all production was communal and planned by the government. Later, from the 1990s on, the land was semi-privatized and herders had the right to use their own land leased from the government; herding became a more individual/domestic activity. Both males and females gained the right to own and fence private land. The involvement of males in both warfare and herding diminished (Zhaoli et al. 2005). Since 2000, all children in the Amdo area were required to attend school, and their future in herding is now uncertain (Sun 2000; Gelek 2006; Beimatsho 2008). All these changes have effects on the local life and will further influence the biased parental investment and stated sex preferences.

Here, we investigate factors that affect real life sex-biased parental investment and stated sex preferences through the following questions: First, do mothers invest more in one or other sex by feeding them longer and longer birth intervals after they are born. Second, do changes over the last 50 years in access to resources, inheritance rules and sex roles influence sex-biased investment. Third, does sex-biased parental investment and/or stated sex preference differ across age groups or between parents of different sex, and do the stated sex preferences reflect the sex-biases observed in parental investment.

## METHODS

This study has approval from Lanzhou University (Life Sciences) and the Research Ethics Committee of University College London.

### Study area

The field work took place between June–October 2014 and March–December 2015. Detailed demographic data were collected in Maqu, located in the eastern part of Tibetan plateau, which stands on the south-west part of Gannan Tibetan autonomous prefecture, Gansu province, China. People in Maqu share a common culture and speak a distinctive dialect of Tibetan (Levine 2014). Over 90% of the population is Tibetan in our study site. Because of limited education resources, most of these Tibetans are herders, selling livestock as a main source of income; some of them also get government benefits as a supplementary income. The marital system used to include polygamy (both polygyny and polyandry) but now is predominately monogamy. In general, the local herders live at 2 sites over the year: one summer site, which is in more remote high altitude areas where families live in yak hair tents, and another winter site, which is more settled and easy to access from local towns and in which the houses are built of mud or bricks (traditional herders moved to many more sites each year). The smallest herding group is called *repkor* (encampment) the composition of which is largely shaped by ecological or kinship factors. The larger herding groups are called *dewa* (tribe) which are generally shaped by cultural relations with neighboring groups and states.

### Demographic data

We collected demography data from 696 households in 5 villages through questionnaires with the help of a local interpreter. Each adult man and woman were interviewed in separate spaces to avoid influence from each other.

### Stated sex preferences

We asked each adult male and female to report their sex preference for offspring at the end of the interview. Some refused to state a preference, but among 654 males and 759 females, 697 individuals (*N* = 330 males and 367 females) reported their sex preference for offspring (mean age = 40.76, SD = 13.29). Young people were less likely to report a preference, probably because they believed it an old fashioned idea to have any sex preference.

### Child mortality

We asked women who had children about their birth history, including those children who were born alive but died later. We also asked about the birth history of their mothers, if their mothers were dead or absent when we did our interview. 1448 women’s birth histories were used in the survival analysis (*N* = 759 direct interviews and 689 indirect interviews, where information was gathered from their children).

We presented data about the marital status to illustrate female economic independence, especially for women who stay single after divorce or being a single mother. Seven types of marital status are included: single mother/father, monogamous marriage, married more than once, unmarried after divorce, unmarried widow/widower, polygynously married, and polyandrously married. All adults were asked about their marital status (*N* = 654 males, *N* = 759 females). We categorized current marital status into single or married in order to investigate whether women prefer to stay single more than men do (see Supplementary Figure 2).

### Infant feeding

For the breastfeeding and bottle-feeding analyses, we interviewed women in our sample that had children after 1990 (*N* = 167), about the start and the end date of breast and bottle feeding. Women who gave birth before 1990 have difficulty remembering the timing of breastfeeding or bottle-feeding, so we excluded them from this analysis to avoid misreporting. We censored children who were still having breast milk at the time we conducted the research (*N* = 220 male children and 191 female children included in the analysis) (Figure 4). We examined the end of breastfeeding and the onset of bottlefeeding within the first 12 months since birth.

### Gift game

We also played an individual gift game with both male and female herders to determine who they would like to give a small amount of money to. By playing a gift giving game, we would like to reveal the social network between households and the different preferences between gift givers. After administering the questionnaire, all the participants are endowed with 15 yuan, in 5 yuan denominations (15 yuan equals approximately 1.8 GB pounds, with which it is possible to buy 4 or 5 500 ml bottles of soft drinks). With 15 yuan at their disposal, they were told they could give 5 yuan to up to 3 people they like. The restrictions were: 1) they cannot give it to anyone within the household, 2) they cannot give it to anyone outside the village, and 3) they cannot keep it themselves. The restriction against giving to others within your own household meant that many parents could not give to their coresident children, but it will not affect giving gifts to any children who have already set up their own family outside of the natal house.

### Statistics

We used cox regression survival analysis to compare the mortality rates for male and female children (Cox 1972). 1448 female birth histories were used in the analysis (which included *N* = 2414 male children and 2212 female children). Those who had had children after 1990 were asked about the duration of breastfeeding and bottlefeeding (*N* = 167 mothers, *N* = 220 male children and 191 female children). Sex differences in mortality before age 5 (Figure 3), the termination of breast-feeding (Figure 4a) and the initiating of bottle-feeding (Figure 4b) were predicted by cox models, controlling for family wealth and parity. To examine the effects of interbirth intervals on sex-biased parental investment, we used cox regression to look at first 3 births and hence the first 2 interbirth intervals. 759 women’s birth histories were used in the cox regression model (*N* = 638 male children, *N* = 554 female children). We used the R package Mumin (Barton 2015) to compare models, including those with and without a sex by cohort interaction, in both the mortality analysis and interbirth interval analysis. The best model was selected based on the lowest Akaike’s Information Criterion.

## RESULTS

### Self-reported sex preferences

**Figure 2:**
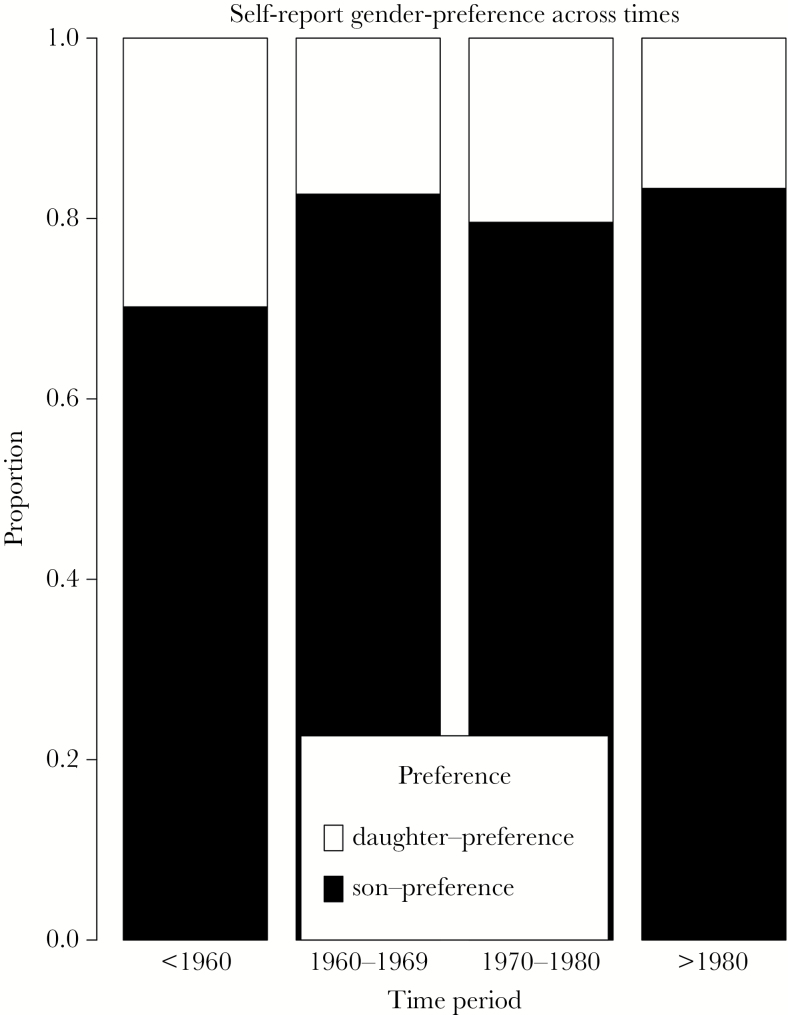
Self-report of offspring sex-preference in 4 different time periods (N = 330 males and *N* = 367 females). The white bar indicates a stated preference for a daughter; the black bar indicates a stated preference for a son.

Self-reported data on the preferred sex of offspring indicates that both men (Supplementary Figure 3) and women (Supplementary Figure 4) who report a preference prefer sons over daughters. Older people show a slightly weaker son preference, but otherwise there is little variation between different age groups and across different time periods (Figure 2). We performed logistic regression to look at whether wealth is associated with sex preference (Supplementary Table 1), controlling for the sex of the reporters. There is no significant difference between male and female stated sex preferences (OR = 0.90, 95% CI = 0.51–1.61, *P* = 0.73). The stated sex preference is statistically different from age group < 29 to age group > 50, with elderly herders more likely to say they prefer daughters than do younger herders (OR = 2.27, 95% CI = 1.29–3.98, *P* = 0.004). Wealthy male reporters say they prefer sons more than do poor individuals, in line with predictions from the Trivers Willard hypothesis (OR = 0.99, 95% CI = 0.99–1.00, *P* = 0.039). For female reporters, there is no effect of wealth (number of yaks) on son preference, and there is no difference between male and female reporters, signified by the nonsignificant interaction (OR = 1.00, 95% CI = 0.99–1.01, *P* = 0.733) (Supplementary Table 1).

### Child mortality before age 5

**Figure 3:**
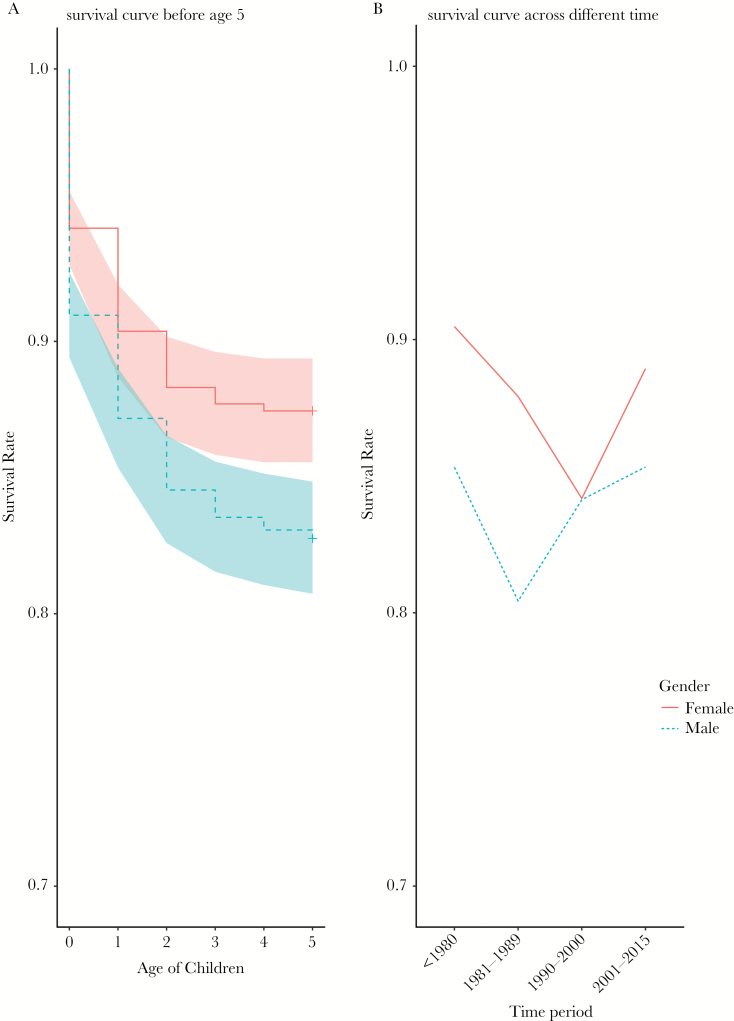
Mortality rate of children (a) before age 5 (b) across different time period. sample includes *N* = 1448 female’s birth history (*N* = 2456 children). Shaded bands indicate 95% confidence intervals. Blue dashed line and shaded band represent survival of male children, red solid line and shaded band represents survival of female children.

We used all 1448 females’ complete birth histories to look at the mortality rate of children before age 5: *N* = 251 male and *N* = 165 female children were reported dead before age 5 (Figure 3a). Figure 3b shows how the survival rate for male and female children differs across different time periods. We conducted a mixed-effects cox model to analyze how ecological factors affect the mortality of children under age 5 controlling for children birth order, sex order, and mother’s age of giving birth (Table 1). Mother’s ID is controlled as random effect in the cox mixed-effects model. Mother’s education and distance to a local clinic do not differ between villages. Only 10 out of 1448 of the women had had an official school education. The villages are close to each other relative to the distance to the clinic, so the distance to the local clinic is the same between villages. The results in Table 1 show that, over the whole-time period, the mortality rate for male children exceeds that of female children (HR = 1.96, 95% CI = 1.32–2.91, *P* < 0.001). Survival rates in different time periods are shown in Table 1. Mothers age at the birth, cohort and offspring sex all influence mortality. A model including an interaction between cohort and sex did not improve the fit of the model (see Supplementary Table 3).

**Table 1:** Mixed-effects Cox model of mortality risk before age 5 (dead = 1, alive = 0)

Variables	HR (95% CI)	*P* value
Fixed effects		
**Son (ref: Daughter**)	**1.96 (1.32–2.91**)	<0.001***
Mother age of giving birth	0.24 (0.13, 0.46)	<0.001***
Children birth year(ref<1980)		
** 1980–1989**	**1.92 (1.28, 2.88**)	0.002**
** **1990–2000	1.25 (0.82, 1.89)	0.29
** **2001–2015	0.98 (0.64, 1.48)	0.91
Yak	1.00 (1.00, 1.00)	0.81
sex order (ref: 1^st^ sex)		
** **2^nd^ sex	1.01 (0.78, 1.32)	0.92
** **3^rd^+ sex	1.16 (0.84, 1.59)	0.37
**Son × Yak**	**0.99 (0.99, 1.00**)	0.04*
Random effects	Variance (SD)	
Mother ID	0.759 (0.86)	

*N* = 2456 children (*N* = 1293 male, *N* = 1163 female) are included in the dataset. Statistical significance indicates in bold, HR stands for Hazards Ratio, 95% CI indicates 95% Confidence intervals.

*P < 0.05, **P < 0.01, ***P < 0.001.

Because nearly all the income of this society is from herding, we consider the number of yaks the most important measure of economic status. There is no association between number of yaks and overall child mortality (see Table 1). However, the sex of children and the number of yaks interact in line with the original formulation of “Trivers-Willard hypothesis”: sons in a wealthy family are relatively more likely to survive than those in a poor family (HR = 0.99, 95% CI = 0.99–1.00, *P* = 0.04). Children who were born in the 1980–1989 cohort had significantly higher mortality from the earlier pre-1980 period (see Table 1).

### Infant feeding

**Figure 4:**
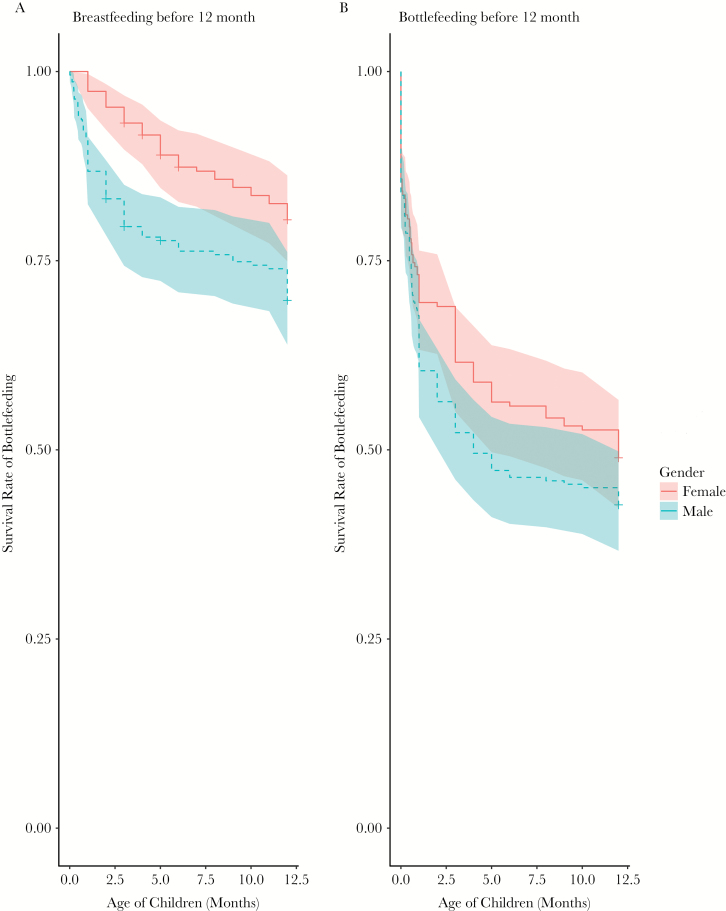
(a) Survival of breastfeeding for children who have been breast fed before 12 months (b) Onset of bottle feeding (shown as the survival rate of exclusive breastfeeding) over 12 months. Sample only includes women who gave birth after 1990 (*N* = 167). The survival curve indicates the likelihood of (a) still being breast fed and (b) starting to be bottle fed by mother before 12 months and 12 months respectively, for male and female children separately. The time was recorded based on days. The shaded bands represent 95% confidence intervals. The blue bands and dashed lines represent male children; the red bands and solid lines represent female children.

The duration of breastfeeding is recognized as a measure of parental investment, especially in the first 12 months when an infant’s nutrition is largely from breast-feeding and when breast-feeding is crucial for the infants’ survival. 66 out of 220 male children and 37 out of 191 female children had stopped breastfeeding before 12 months (Figure 4a). The mean length for breastfeeding for female children is 10.62 months and for male children is 9.36 months; Cox regression indicated this is a statistically significant sex difference (HR = 1.738, 95% CI = 1.162–2.599, *P* = 0.007).

Mothers initiate bottle-feeding as a way of supplementing breast milk. 97 female children and 125 male children started bottle-feeding before 12 months (Figure 4b). The mean time for female children to start bottle-feeding is 7.12 months, and for male children is 5.98 months. Cox regression showed a small sex difference in the duration of exclusive breastfeeding (i.e., the earlier introduction of bottle feeding) (HR = 1.212, 95% CI = 0.93–1.58, *P* = 0.156).

### The length of the birth interval

**Figure 5:**
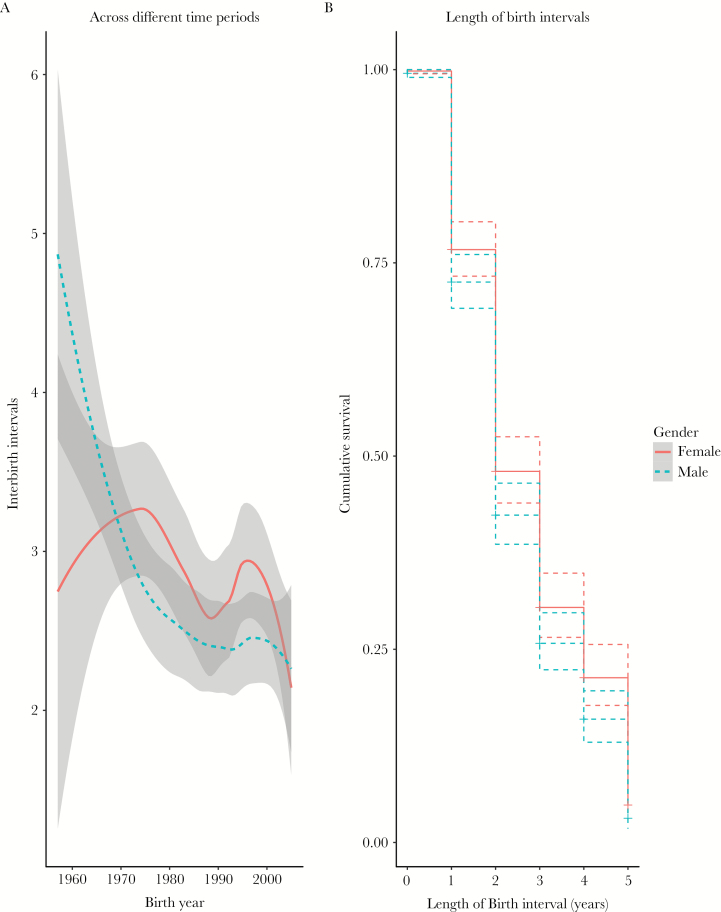
(a) Interbirth intervals across different time periods in 2 sex groups. The red solid line represents interbirth intervals after a female child; the blue dashed line represents interbirth intervals after a male child. The shaded bands represent 95% confidence intervals. (b) The survival function of the birth intervals for mothers after having a son (blue line) and after a daughter (red solid line) over the whole dataset.

The nationwide family planning policy was implemented in this area in the 1980s; from that time on, every family in this area was allowed no more than 3 children. Figure 5a shows the birth interval after a son was longer than after a daughter before 1970, but after 1970 the intervals became longer after a daughter. After 2000, when the compulsory primary education was introduced, every family had to send school-age children to a local boarding school, and sex-biased parental investment seems to decline, at least in terms of the interbirth intervals (Figure 5a). However, a model including an interaction between cohort and sex did not improve the fit of the model (see Supplementary Table 4). Over all the data, which covers the last 50 years, Figure 5b shows the length of the IBI after having a son is significantly shorter than after having a daughter (Table 2), but we do not find any evidence for TWH for the birth interval analysis.

**Table 2:** The effects of sex and different time period on the length of the interbirth intervals by using Cox regression

Variables	HR (95% CI)	*P* value
Sex (ref: Daughter)		
** Son**	**1.25 (1.02–1.54**)	0.03*
Mother’s age of giving birth	0.97 (0.95–0.99)	<0.001***
Children birth year (ref:<1980)		
** **birth year 1980–1989	1.42 (1.11–1.81)	0.01**
** **birth year 1990–2000	1.69 (1.36–2.09)	<0.001***
** **birth year 2001–2015	2.44 (1.99–3.00)	<0.001***
Yak	1.00 (1.00–1.00)	0.79
Sex order (ref: 1^st^ sex)		
** **2^nd^ sex	0.48 (0.40–0.59)	<0.001***
Son × Yak	1.00 (1.00–1.00)	0.89

HR stands for Hazard Ratio, 95% CI indicates 95% confidence intervals.

*P < 0.05, **P < 0.01, ***P < 0.001.

Table 2 shows that over the whole period up to 2010, interbirth intervals after a boy were significantly shorter than after a girl (rate of birth HR = 1.25, 95% CI = 1.02–1.54, *P* = 0.03); the birth intervals across different time periods (cohort) and sex order (whether same sex as previous birth) are strongly associated with the length of the birth interval. Other covariates included mother’s age of giving birth and the number of livestock. Birth intervals became shorter and shorter from the 1980s until after 2000, in which time period the compulsory education was implemented. Sex order indicated a strong effect on the risk of finishing reproduction, in that the length of interbirth interval is reduced if the second born is the same sex as the first one (HR = 0.48, 95% CI = 0.40–0.59, *P* < 0.001), suggesting a preference for having children of both sexes.

### Gift decisions

We used a gift game to examine how gifts to direct kin (parents, sibling, offspring) depend on the sex of the giver and the receiver. In the gift game, there are 150 male givers and 157 female givers giving gifts to direct kin. Table 3 shows that the sex of the giver strongly determines which parent is given a gift: same-sex offspring give significantly more gifts to same-sex parents (chi square = 12.14, *P* < 0.001). This suggests that mothers may be correct in assuming that daughters are more likely to help support them in adulthood (whereas fathers may be more likely to get help from sons). Regarding offspring, mothers are more likely to give gifts to daughters than are fathers (chi square = 3.11, *P* = 0.08). As the mean age of women who took part in the gift-giving game was 41.67, and because the restriction of the game that givers are not allowed to give gifts to the individuals living in the same household, the possibility of giving gifts to offspring was low for younger parents.

**Table 3:** The patterns of giving gifts to direct kin by adult male and female herders

	Male giver	Female giver	Chi square	*P* value
Father	33	19	12.14	<0.001
Mother	13	33		
**Sibling**				

	Male giver	Female giver	Chi square	*P* value
Brother	45	38	0.93	0.34
Sister	32	37		
**Children**				

	Male giver	Female giver	Chi square	*P* value
Son	15	12	3.11	0.08
Daughter	12	24		

Results of 2 × 2 Chi-square test indicate statistical difference between male and female givers preferentially giving to same sex kin. The results are divided into gifts to parents, siblings and children.

## DISCUSSION

Levine (1987) reported that most Tibetan societies have son-biased parental investment. From our self-report data, male and female herders report this norm as their current preference. However, we find that this stated son preference did not reflect the behavioral measures taken by mothers to invest in their children over the last 50 years.

The birth interval and mortality data both suggest that 1980–1990 reflected a period of increased mortality, followed by a steady decline in mortality. Birth rates sped up throughout this period. All our measures suggest mothers in our study area are generally investing more in daughters. Data on all births from the last 50 years suggested that daughters are generally more likely to survive than sons over their first 5 years. Feeding data shows that daughters were being breastfed for longer and sons were put onto milk substitutes earlier, indicating a female-biased parental investment in terms of nutrition decisions. The longitudinal data from children born in different age cohorts show that there is generally a longer interbirth interval after a daughter.

The marital status data suggested low levels of polygamous marriage in this pastoralist society, although polygamous mating opportunities are still present. There are significantly more female than male single parents (see Supplementary Figure 2), which we interpret as demonstrating increased female economic independence, due to the relatively higher economic and labour contributions to the household from females (Gates 1993; Hare 1999). Gift giving decisions also suggested stronger investments in same-sex offspring, especially by females. As children get older, reciprocity may be at play, given that daughters were more likely to give gifts to their mother, and in Tibetan society daughters are generally more helpful in looking after elderly parents (Childs et al. 2011).

Policies over the last 50 years have had an impact on sex roles and rights that we believe influenced sex-biased investment. During the early stages of the socialist regime, livestock was owned communally, individual decision-making was limited, and dowry and brideprice were not allowed as animals were not the property of individuals. In the period of collectives and communes (see SI), resources were equally distributed, and the property and food each family received were largely dependent on the number of labourers in the household, so having a big family ensured more income (Chen 2004). When wealth inequality was low, men did not have the same potential for polygyny. The new roles and high workloads women adopted within the family diminished males’ contribution to the domestic economy. After 1990, the privatization policy marked a major changing point in sex-biases in wealth. Shares of the grassland were allocated to each household according to the number of people in their family and most plots were fenced. The land was given to families for 50 years of use, although no system of inheritance was made clear. Women were allowed to have their own portion of land to use. When they married, women could take their own property with them in the form of dowry. But because land is not moveable, women tended to rent their land back to their parents or siblings at their natal house, or she could use the land herself, especially when her husband’s land is not enough for their livestock. The restrictions on land use and the equal opportunity of land inheritance affected the sex-biased dispersal pattern at marriage, with women dispersing less. Currently, few individuals migrate into the county (8% male, 4% female are from outside of the county) and there is little difference between the number of males and females born in the village (see Supplementary Figure 1). Fencing practices, adopted in the 1990s, largely reduced the need for men to herd animals actively, which reduced male workloads; female workloads increased, however, as the distance to water often increased, and the necessity of growing barley to feed animals also increased because pasture space after the introduction of fencing has shrunk and some animals were in danger of malnutrition (Yan et al. 2011). The shift towards higher female workloads was likely to underlie the increase in daughter-biased investment as daughters became more helpful at enhancing wealth and more likely to bring benefits to their mothers (Gowaty and Lennartz 1985; Gates 1993; Margulis et al. 1993; Pirie 2005; Zhaoli et al. 2005). Since the introduction of the 9-year compulsory education system, all children were sent to boarding schools after 2000, for the sake of “Pastures to Grassland” (Yeh 2005). As these children are likely to abandon herding in the future after education (Bessho 2015), there are no longer likely to be predictable differences between the sexes in terms of potential on the job market.

Self-reporting is the general method of measuring sex preference used by demographers (Murphy et al. 2011), which we also used here, and compare it with biodemographic data on mortality, parental investment and feeding. The majority of the respondents reported that they preferred sons over daughters, so the self-report results on sex preference do not match the detailed data on sex-biased parental investment, at least with respect to maternal investment. However one area where self-report matched investment was with respect to the interaction between wealth (in yaks) and son preference. Both mortality data and the stated preferences of males and females showed that wealthy families with many yaks stated a stronger preference for males and showed higher survival of males relative to females than poorer families (in other words females were relatively more likely to survive and be preferred in poorer families).

The mismatch between daughter-biased maternal investment and stated son preference suggests individuals continue to report behavior as fitting longstanding social norms, which in this case is to prefer sons. Norms, generally defined as shared cultural preferences guiding the behavior of group members, may be slower to change than the costs and benefits that have more immediate influences on actual behavior. In this case, male norms and behavior may not show this mismatch, as males are not involved in infant feeding decisions and in the gift game they favored male recipients, so it may only be females who stated norms that are not reflected in their behavior. Females may incur reputational costs if they were to change their stated norms from deep-rooted cultural beliefs, or they may simply internalize those norms and give standard responses when asked. It should be noted that China has put out much propaganda against having any sex preference since the implementation of its one-child policy in the1980s, so that may also explain why a large proportion of the sample did not want to state any preference. However, this is not the only case where stated sex preferences for males do not reflect behavior. Cronk (1993), who showed that the Kenyan Mukogodo favored daughters in their parental investment decisions, also comments on the fact that their stated preference was for sons. There are many examples in other domains showing that there is an inconsistency in individual’s actual behavior and their intention when reputation is at stake (e.g., some lab-based experiments showing that individuals show widely acknowledged “good characters” when they think that there were possible mating opportunities [Barclay 2010]). An example where most individuals’ documented behavior does not follow their reported preference is cases where stated fertility preference systematically exceeds actual family size (Cypriot et al. 2010). Another example from Grosjean and Brooks (2017) indicates that historical regional differences in sex ratios still appear to influence attitudes today even generations after the imbalance in sex ratios had disappeared.

The evolutionary processes leading to the emergence of cultural norms may generate some conflicts of interest between individuals and their family or the wider group. For example, Micheletti et al. (2017) have shown, through models that maximize inclusive fitness, that there can be different interests in males and females and in the older generation and the younger generation, leading to possible parent-offspring and parent-parent conflict in favored behaviors. They show how the older generation is predicted to favor warlike behavior in young men, to protect the extended family, whereas younger generation males and females could benefit from less warfare and more from individualistic interests. It is possible that cultural evolutionary processes generate stated norms (or ideals) that favor the group, or favor the more powerful members of the group, but these do not necessarily reflect variations in individual behavior, especially that of less dominant members of the group such as women. When answering a question on sex preference, both men and women may be reiterating long accepted cultural norms. But in day-to-day parental care, mothers appear to be responding to more immediate changes in individual costs and benefits that would benefit their own inclusive fitness.

## SUPPLEMENTARY MATERIAL

Supplementary data are available at *Behavioral Ecology* online.

## FUNDING

This work was supported by the China Scholarship Council and Research Council of Norway (grant No. 240280) and by the 1000 Talent Plan of China and Lanzhou University.

## AUTHOR CONTRIBUTIONS

R.M. and J.D. designed the study. J.D. collected and analyzed the data. J.D. and R.M. wrote the paper.

Data availability: Analyses reported in this article can be reproduced using the data provided by Du and Mace (2017).

## Supplementary Material

Supplementary Material

## References

[CIT0001] Aitane I 2009 The determinants of discrimination against daughters in China: evidence from a provincial-level analysis. Routledqe Population Studies. 63:41–14. 10.1080/00324720802535023 19184723

[CIT0002] Alexander RD 1974 The evolution of social behavior. Annu Rev Ecol Evol Syst. 5:325–383.

[CIT0003] Anderson KH, King EM, Wang Y 2003 Market returns, transfers and demand for schooling in Malaysia, 1976–89. J Dev Stud. 39:1–28.

[CIT0004] Banister J 2004 Shortage of girls in China. J Popul Res. 21:19–45.

[CIT0005] Barclay P 2010 Altruism as a courtship display: some effects of third-party generosity on audience perceptions. Br J Psychol. 101:123–135. 19397845 10.1348/000712609X435733

[CIT0006] Barton K 2015 Multi-model inference. version 1.15.6, package.

[CIT0007] Beimatsho 2008 Population, pasture pressure, and school education: case studies from Nag chu, TAR, PRC. JIATS, no. 4 (December 2008).

[CIT0008] Bereczkei T, Dunbar RIM 1997 Female-biased reproductive strategies in a Hungarian Gypsy population. Proc R Soc Lond B Biol Sci. 264:17–22.

[CIT0009] Bessho Y 2015 Migration for ecological preservation? Tibetan herders’ decision making process in the eco-migration policy of Golok Tibetan autonomous prefecture (Qinghai province, PRC). Nomadic Peoples. 19:189–208.

[CIT0010] Bezner Kerr R, Dakishoni L, Shumba L, Msachi R, Chirwa M 2008 “We grandmothers know plenty”: breastfeeding, complementary feeding and the multifaceted role of grandmothers in Malawi. Soc Sci Med. 66:1095–1105. 18155334 10.1016/j.socscimed.2007.11.019

[CIT0011] Chen F 2004 The division of labor between generations of women in rural China. Soc Sci Res. 33:557–580.

[CIT0012] Childs G, Goldstein MC, Wangdui P 2011 Externally-resident daughters, social capital, and support for the elderly in rural Tibet. J Cross Cult Gerontol. 26:1–22. 21234664 10.1007/s10823-010-9135-5

[CIT0013] Cox DR 1972 Regression Models and Life-Tables. J R Stat Soc Series B Stat Methodol. 34:187–220.

[CIT0014] Crognier E, Villena M, Vargas E 2002 Helping patterns and reproductive success in Aymara communities. Am J Hum Biol. 14:372–379. 12001095 10.1002/ajhb.10047

[CIT0015] Cronk L 1989 Low socioeconomic status and female-biased parental investment: the Mukogodo example. Am Anthropol. 91:414–429.

[CIT0016] Cronk L 1991 Preferential parental investment in daughters over sons. Hum Nat. 2:387–417. 24222341 10.1007/BF02692198

[CIT0017] Cronk L 1993 Parental favoritism toward daughters. Am Sci. 81:272–279.

[CIT0018] Cypriot G, Republic T, Nations U, Union E, States M, Commission E, Republic T 2010 OECD family database. Social Policy, 1–5.

[CIT0019] Degraff DS, Bilsborrow RE 1996 Southern demographic association children’s education in the Phillipines : does high fertility matter ? Popul Res Policy Rev. 15:219–247.

[CIT0020] Dewey KG 1998 Cross-cultural patterns of growth and nutritional status of breast-fed infants. Am J Clin Nutr. 67:10–17. http://www.ncbi.nlm.nih.gov/pubmed/9440369. 9440369 10.1093/ajcn/67.1.10

[CIT0021] Dunbar RIM 2002 Helping-at-the-nest and sex-biased parental investment in a Hungarian Gypsy population. Curr Anthropol. 43:804–809.

[CIT0022] Du J, Mace R 2017 Data from: parental investment in a Tibetan population does not reflect stated cultural norms. Dryad Digital Repository. http://dx.doi.org/10.5061/dryad.d3ns1 10.1093/beheco/arx134 PMC5873243 29622933

[CIT0023] Edlund L 1999 Son preference, sex ratios, and marriage patterns. J Political Econ. 107:1275–1278.

[CIT0024] Fujita M, Roth E, Lo YJ, Hurst C, Vollner J, Kendell A 2012 In poor families, mothers’ milk is richer for daughters than sons: a test of Trivers-Willard hypothesis in agropastoral settlements in Northern Kenya. Am J Phys Anthropol. 149:52–59. 22623326 10.1002/ajpa.22092

[CIT0025] Gates H 1993 Cultural support for birth limitation among urban capital-owning women. In: Davis D, Harrell S, editors. Chinese families in the post-mao era. Berkeley: University of California Press pp. 151–274.

[CIT0026] Gaulin SJC, Boster JS 1990 Dowry as female competition. Am Anthropol. 92:994–1005.

[CIT0027] Gelek L 2006 Anthropological field survey on basic education development in the Tibetan nomadic community of Maqu, Gansu, Anthropological Field Survey on Basic Education Development in the Tibetan Nomadic Community of Maqu, Gansu, China. Asian Ethnicity. 7:37–41.

[CIT0028] Gowaty PA, Lennartz MR 1985 Sex ratios of nestling and fledgling red-cockaded woodpeckers (*Picoides borealis*) favor males. American Society of Naturalists. 126:347–353.

[CIT0029] Grosjean P, Brooks RC 2017 Persistent effect of sex ratios on relationship quality and life satisfaction. Phil Trans R Soc B. 372:1–24. 10.1098/rstb.2016.0315 PMC5540857 28760758

[CIT0030] Hamilton WDD 1964 The genetical evolution of social behaviour. I. J Theor Biol. 7:1–16. 5875341 10.1016/0022-5193(64)90038-4

[CIT0031] Hare D 1999 Women’s economic status in rural China: household contributions to male-female disparities in the wage-labor market. World Dev. 27:1011–1029.

[CIT0032] Hartung J 1976 On natural selection and the inheritance of wealth. Curr Anthropol. 17:607.

[CIT0033] He QQ, Wu JJ, Ji T, Tao Y, Mace R 2016 Not leaving home: grandmothers and male dispersal in a duolocal human society. Behav Ecol. 27:1343–1352. 27656085 10.1093/beheco/arw053 PMC5027622

[CIT0034] Henrich J, Boyd R 2001 Why people punish defectors. Weak conformist transmission can stabilize costly enforcement of norms in cooperative dilemmas. J Theor Biol. 208:79–89. 11162054 10.1006/jtbi.2000.2202

[CIT0035] Henrich J, Broesch J 2011 On the nature of cultural transmission networks: evidence from Fijian villages for adaptive learning biases. Philos Trans R Soc Lond B Biol Sci. 366:1139–1148. 21357236 10.1098/rstb.2010.0323 PMC3049092

[CIT0036] Holden CJ, Sear R, Mace R 2003 Matriliny as daughter-biased investment. Evol Hum Behav. 24:99–112.

[CIT0037] Huber T 2012 The changing role of hunting and wildlife in pastoral communities of Northern Tibet. In: Kreutzmann H, editor. Pastoral practices in High Asia. Netherlands: Springer p. 195–215.

[CIT0038] Kalanda BF, Verhoeff FH, Brabin BJ 2006 Breast and complementary feeding practices in relation to morbidity and growth in Malawian infants. Eur J Clin Nutr. 60:401–407. 16306929 10.1038/sj.ejcn.1602330

[CIT0039] Keller MC, Nesse RM, Hofferth S 2001 The Trivers – Willard hypothesis of parental investment No effect in the contemporary United States. Evol Hum Behav. 22:343–360.

[CIT0040] Kramer KL 2010 Cooperative breeding and its significance to the demographic success of humans. Ann Rev Anthropol. 39:417–436.

[CIT0041] Levine NE 1987 Differential child care in three tibetan communities: beyond son preference. Popul Dev Rev. 13:281–304.

[CIT0042] Levine NE 2014 Reconstructing tradition: persistence and change in golog social structure. Available online: www.cwru.edu/affil/tibet/booksAndPapers/ Segpaper.htm

[CIT0043] Levine NE 2015 Transforming inequality: eastern Tibetan pastoralists from 1955 to the present. Nomadic Peoples. 19:164–188.

[CIT0044] Mace R 1996 Biased parental investment and reproductive success in Gabbra pastoralists. Behav Ecol Sociobiol. 38:75–81. 12292074 10.1007/s002650050219

[CIT0045] Mace R 2010 Ch 15. Social behaviour in humans. In: Székely T, Moore AJ, Komdeur J, editors. Social behaviour: genes, ecology and evolution C.U.P. Vol. 177 p. 395–409.

[CIT0046] Mace R, Sear R 1997 Birth interval and the sex of children in a traditional African population: an evolutionary analysis. J Biosoc Sci. 29:499–507. 9881149 10.1017/s0021932097004999

[CIT0047] Margulis SW, Altmann J, Ober C 1993 Sex-biased lactational duration in a human population and its reproductive costs. Behav Ecol Sociobiol. 32:41–45. 12286204 10.1007/BF00172221

[CIT0048] Martin MA, Garcia G, Kaplan HS, Gurven MD 2016 Conflict or congruence? Maternal and infant-centric factors associated with shorter exclusive breastfeeding durations among the Tsimane. Soc Sci Med. 170:9–17. 27732906 10.1016/j.socscimed.2016.10.003 PMC5107317

[CIT0049] Mattison SM, Beheim B, Chak B, Buston P 2016 Offspring sex preferences among patrilineal and matrilineal Mosuo in Southwest China revealed by differences in parity progression. R Soc Open Sci. 3:160526. doi:10.1098/rsos.160526 27703713 10.1098/rsos.160526 PMC5043333

[CIT0050] Mattison SM, Wander K, Hinde K 2015 Breastfeeding over two years is associated with longer birth intervals, but not measures of growth or health, among children in Kilimanjaro, TZ. Am J Hum Biol. 27:807–815. 25945696 10.1002/ajhb.22729

[CIT0051] Mesoudi A, Chang L, Murray K, Lu HJ 2015 Higher frequency of social learning in China than in the West shows cultural variation in the dynamics of cultural evolution. Proc R Soc B. 282:1–7. 10.1098/rspb.2014.2209 PMC4262178 25392473

[CIT0052] Micheletti AJC, Ruxton GD, Gardner A 2017 Intrafamily and intragenomic confilcts in human warfare. Proc R Soc B. 284:20162699. doi:10.1098/rspb.2016.2699 10.1098/rspb.2016.2699 PMC5326533 28228515

[CIT0053] Murphy R, Tao R, Lu X 2011 Son preference in rural China: patrilineal families and socioeconomic change. Popul Dev Rev. 37:665–690. 22319769 10.1111/j.1728-4457.2011.00452.x

[CIT0054] Oddy W 2001 Breastfeeding protects against illness and infection in infants and children: a review of the evidence. Breastfeeding Review. 9:11–18. http://search.ebscohost.com/login.aspx?direct=true&db=cin20&AN=106929140&site=ehost-live. 11550600

[CIT0055] Pirie F 2005 Segmentation within the state: the reconfiguration of Tibetan Tribes in China’s reform period. Nomadic Peoples. 9:83–102.

[CIT0056] Sieff DF, Betzig L, Cronk L, Fix AG, Flinn M, Gibson K, Sattenspie L, Herring DA, Howell N, Sieff DF 1990 Explaining biased sex ratios in human populations. Curr Anthropol. 31:33–34.

[CIT0057] Sparks CS 2011 Parental investment and socioeconomic status influences on children’s height in Honduras: an analysis of national data. Am J Hum Biol. 23:80–88. 21080444 10.1002/ajhb.21104

[CIT0058] Sun Z 2000 A study of education problems in Tibetan pastoral areas. Journal of Central University for Nationalities (Philosophy and Social Science). 27:83–90.

[CIT0059] Trivers RL 1972 Parental investment and sexual selection. In: Campbell B, editor. Sexual selection and the descent of man. Chicago: Aldine p. 136–179.

[CIT0060] Veller C, Haig D, Nowak MA 2016 The Trivers–Willard hypothesis: sex ratio or investment?Proc R Soc B: Biol Sci. 283:1–9. 10.1098/rspb.2016.0126 PMC4874707 27170721

[CIT0061] Wander K, Mattison SM 2013 The evolutionary ecology of early weaning in Kilimanjaro, Tanzania. Proc R Soc Lond B Biol Sci. 280:20131359. 10.1098/rspb.2013.1359 PMC3757970 23926151

[CIT0063] WHO 2003 Global strategy for infant and young child feeding. Geneva, Switzerland: World Health Organization, 1–30. https://doi.org/ISBN 92 4 156221 8

[CIT0064] Wilhelm SL, Rodehorst TK, Stepans MB, Hertzog M, Berens C 2008 Influence of intention and self-efficacy levels on duration of breastfeeding for midwest rural mothers. Appl Nurs Res. 21:123–130. 18684405 10.1016/j.apnr.2006.10.005

[CIT0065] Williamson NE 1976 Sons or daughters a cross-cultural survey of parental preferences1st ed London: Sage Publication.

[CIT0066] Yan J, Wu Y, Zhang Y 2011 Adaptation strategies to pasture degradation: gap between government and local nomads in the eastern Tibetan Plateau. Journal of Geographical Sciences. 21:1112–1122.

[CIT0067] Yeh ET 2003 Tibetan range wars: spatial politics and authority on the grasslands of Amdo. Development and Change. 34:499–523.

[CIT0068] Yeh ET 2005 Green governmentality and pastoralism in western China: “Converting Pastures to Grasslands.” Nomadic Peoples. 9:9–30.

[CIT0069] Yeh ET, Nyima Y, Hopping KA, Klein JA 2014 Tibetan pastoralists’ vulnerability to climate change: a political ecology analysis of capacity to cope with snowstorms. Hum Ecol. 42(1):61–74. doi:10.1007/s10745-013-9625-5

[CIT0070] Zhan HJ, Montgomery RJV 2003 Gender and elder care in China: the influence of filial piety and structural constraints. Gender and Society. 17:209–229.

[CIT0071] Zhaoli Y, Ning W, Dorji Y, Jia R 2005 A review of rangeland privatisation and its implications in the Tibetan Plateau, China. Nomadic Peoples. 9:31–51.

