# Supplementary material for: Parental investment in Tibetan populations does not reflect stated cultural norms
Source: Behav Ecol. 2017 Oct 20;29(1):106–16. doi: 10.1093/beheco/arx134 (PMC5873243; doi:10.1093/beheco/arx134)
Supplement: Supplementary Material [file arx134_suppl_supplementary_information.docx]

**Supplementary information for**

**Parental investment in Tibetan populations does not reflect stated cultural norms**

**Juan Du & Ruth Mace**

**Major political changes 1950-2000:**

In recent history, pastoralists were nomadic and kept searching for better grazing land, and those groups with strong leadership and effective fighting forces gained better land (Levine, 2015). In the twentieth century, pastoralists on the Tibetan plateau and nearby areas underwent a series of political changes. Policies which related to the livestock and grassland in the twentieth century included what we describe as Collectives, Communes, Household Responsibility system, Privatization, and Settlement action (See below). Policies related to the cultural life in this time period included the child policy and the introduction of a formal education system. We think that all these policies affected the sex preference in the society in various ways helping shift gender preference away from sons toward daughters. We will briefly introduce the major policies which were implemented in this area from earlier 1950s until 2000.

**Communist regime**: after the establishment of the People’s Republic of China in the earlier 1950’s, livestock were redistributed among households with the purpose of reducing wealth inequality between rich and poor (Goldstein & Beall, 1991). This policy marks the beginning of a programme of major interventions in the pastoralist system (Wu & Richard, 1999; Levine, 2015). Between 1958 and 1960 there was serious starvation in the area, as there was all over China at that time, associated with the aftermath of Mao’s ‘Great Leap Forward’.

**Collective**: Collective was established in 1964; at that time pastoralist from different classes were forced to herd together in groups, and a “points” system was introduced. How many points each person got at the end of the year depended on the difficulty and workload he/she accomplished. The allocation of resources to each household in each community was a share of the collective’s production calculated from two parts: forty percent depended on the total points, and sixty percent depended on the original wealth of each household (Zhaoli et al., 2005; Wu & Richard, 1999; Miller, 2000; Zhaoli et al., 2005).

**Commune**: Commune was introduced in 1968. At that time, all animals belonged to the commune, and pastoralists shared their production each year. The share received was based on two parts, forty percent of a number of family member, sixty percent of the total points (Clarke, 1998; Gruschke, 2012; Huber, n.d.; Miller, 1999; Pirie, 2005). China was undergoing the Cultural Revolution during this period.

**Household responsibility**: In 1981, livestock were divided among every family, but the land was open access. This was said to have caused overgrazing and grassland degradation as is often associated with using common pool resources (Goldstein & Beall, 1991; Hardin, 1968).

**Privatisation**: The privatisation policy was first introduced in the mid-1980s in Qinghai Province, and quickly spread to the neighbouring province of Gansu where the field site in Maqu is located (Miller, 2000). In the early 1990s, privatisation was implemented to confront the problems of grassland degradation and communal access, and the government began to settle the nomads. First in the winter pasture, and later in summer pastures too, rangeland was divided between each household based on how many people are in each household, and each family allocated a rangeland for 50 years of use, with little or no instructions given on land inheritance policy (Yamaguchi, 2011;Williams, 1996;Wu & Richard, 1999).

**Sedentarisation**: In 2000, the “Great Development of the West” campaign was launched in the western regions of China aimed at improving the infrastructure and the living standard of people in this area. One of the very important ways of achieving the development was thought to be sedentarisation so there were significant moves to settle nomads. The Government tried to settle the herders in two ways. The first is to settle herders in their winter grassland, the second was to move herders into newly-built towns. The first method changes the herder from a highly mobile into a semi-settled lifestyle, and the latter strategy tried to change the source of herder’s income from livestock to a more market-oriented economy ( Gruschke, 2008; Gyal, 2015; Levine, 2015; Ptackova, 2011, 2012, 2015; Yeh, 2005). In Maqu, herders started to move into settled houses from 2003 onward (Levine, 2015).

**Child policy**: In 1979, the ‘one child policy’ was first implemented in the Han areas, and was extended to ethnic minority groups in the late 1980s (Attané, 2002). There were differences between Han and other ethnic groups not only differences in timing in different parts of China but also based on the rural or urban nature of an area (Guo Zhigang, 2003; Attané, 2002). According to the provincial family planning regulations in 1990, urban Tibetans were allowed to have 2 to 3 children, but no restriction was imposed on the rural Tibetans at that time. Urban Chinese Tibetans were allowed to have 2 whereas rural Chinese Tibetan were later restricted to have 3 (Attané & Courbage, 2000; Melvyn C ., Goldstein, 1991). Our own data show that in Maqu people were allowed to have three children after late 1980s to the present day.

**Education**: In 2000, the ‘Great Western Development policy’ was strengthened by giving priority to the construction of schools, especially for the basic education system in minority groups in the western regions. In Maqu, the government started to build boarding schools in the late 90s; there are altogether 14 schools in the county. By the year of 2000, the enrolment rate had risen significantly compared to very low rates of secondary education before (Gelek, 2006).

**SI References**

Attané, I. (2002). China’s Family Planning Policy: An Overview of Its Past and Future. *Studies in Family Planning*, *33*(1), 103–113.

Attané, I., & Courbage, Y. (2000). Transitional stages and identity boundaries: The Case of Ethnic Minorities in China. *Population and Environment*, *21*(3), 257–280. https://doi.org/10.1007/BF02436131

Clarke, G. E. (1998). Development, Society, and Environment in Tibet. In *Proceedings of the 7th seminar of the International Association for Tibetan Studies*.

Gelek, L. (2006). Anthropological field survey on basic education development in the Tibetan nomadic community of Maqu , Gansu, Anthropological Field Survey on Basic Education Development in the Tibetan Nomadic Community of Maqu , Gansu, China. *Asian Ethnicity*, *7*(April 2014), 37–41. https://doi.org/10.1080/14631360500505777

Goldstein, M. C., & Beall, C. M. (1991). Change and Continuity in Nomadic Pastoralism on the Western Tibetan plateau. *Nomadic Peoples*, (28).

Gruschke, A. (2008). International Association of Tibetan Studies, (4).

Gruschke, A. (2012). Tibetan Pastoralists in Transition. Political Change and State Interventions in Nomad. In H. Kreutzmann (Ed.), *Pastoral practices in High Asia* (1st ed., pp. 273–289). Dordrecht: Springer. https://doi.org/10.1007/978-94-007-3846-1

Guo Zhigang. (2003). Cong Zheng Ce Sheng Yu Kan Zhong Guo Sheng Yu Zheng Ce de Duo Yang Xing. *Population Research*, *27*(5).(in Chinese)

Gyal, H. (2015). The politics of standardising and subordinating subjects: the nomadic settlement project in Tibetan areas of Amdo. *Nomadic Peoples*, *19*(2), 241–260. https://doi.org/10.3197/np.2015.190206

Hardin, G. (1968). The tragedy of the commons. *Science*, *162*(3859), 1243–8. https://doi.org/10.1126/science.162.3859.1243

Huber, T. (n.d.). The Changing Role of Hunting and Wildlife in Pastoral Communities of Northern Tibet, 195–215. https://doi.org/10.1007/978-94-007-3846-1

Melvyn C . Goldstein, C. M. B. (1991). China Birth Control Policy in the Tibet Autonomous Region : Myths and Realities. *Asian Survey*, *31*(3), 285–303.

Miller, D. J. (1999). Nomads of the Tibetan Plateau Rangelands in Western China — Part Three : Pastoral Development and Future Challenges. *Rangelands*, *21*(2), 17–20.

Miller, D. J. (2000). Tough Times for Tibetan Nomads in Western China: Snowstorms, Settling down, Fences and the Demise of Traditional Nomadic Pastoralism. *Nomadic Peoples*, *4*(1), 83–109. https://doi.org/10.3167/082279400782310674

Pirie, F. (2005). Segmentation Within the State: The Reconfiguration of Tibetan Tribes In China’s Reform Period. *Nomadic Peoples*, *9*(1), 83–102. https://doi.org/10.3167/082279405781826074

Ptackova, J. (2011). Sedentarisation of Tibetan nomads in China: Implementation of the Nomadic settlement project in the Tibetan Amdo area; Qinghai and Sichuan Provinces. *Pastoralism: Research, Policy and Practice*, *1*(1), 4. https://doi.org/10.1186/2041-7136-1-4

Ptackova, J. (2012). Implementation of Resettlement Programmes Amongst Pastoralist Communities in Eastern Tibet. In H. Kreutzmann (Ed.), *Pastoral practices in High Asia* (pp. 217–234). Dordrecht: Springer Netherlands. https://doi.org/10.1007/978-94-007-3846-1

Ptackova, J. (2015). Hor - a sedentarisation success for Tibetan pastoralists in Qinghai? *Nomadic Peoples*, *19*(2), 221–240. https://doi.org/10.3197/np.2015.190205

Williams, D. E. E. M. (1996). Grassland Enclosures: Catalyst of Land Degradation in Inner Mongolia. *Human Organization*, *55*(3), 307–313.

Wu, N., & Richard, C. (1999). The privatisation process of rangeland and its impacts on pastoral dynamics in the Hindu- Kush Himalaya: The Case of Western Sichuan, China. *International Rangeland Congress, Townsville Australia*, 1–17.

Yamaguchi, T. (2011). The transition of Mountain Pastoralism: An Agrodiversity Analysis of the Livestock Population and Herding Strategies in Southeast Tibet, China. *Human Ecology*, *39*(2), 141–154. https://doi.org/10.1007/s10745-010-9370-y

Yeh, E. T. (2003). Tibetan Range Wars: Spatial Politics and Authority on the Grasslands of Amdo. *Development and Change*, *34*(3), 499–523. https://doi.org/10.1111/1467-7660.00316

Yeh, E. T. (2005). Green Governmentality and Pastoralism in Western China: “Converting Pastures to Grasslands.” *Nomadic Peoples*, *9*(2005), 9–30. https://doi.org/10.3167/082279405781826164

Yeh, E. T., Nyima, Y., Hopping, K. A., & Klein, J. A. (2014). Tibetan Pastoralists’ Vulnerability to Climate Change: A Political Ecology Analysis of Snowstorm Coping Capacity. *Human Ecology*, *42*(1). https://doi.org/10.1007/s10745-013-9625-5

Zhaoli, Y., Ning, W., Dorji, Y., & Jia, R. (2005). A Review of Rangeland Privatisation and its Implications in the Tibetan Plateau, China. *Nomadic Peoples*, *9*(1), 31–51. https://doi.org/10.3167/082279405781826155

**Supplementary tables and figures**

**Dispersal at marriage:**


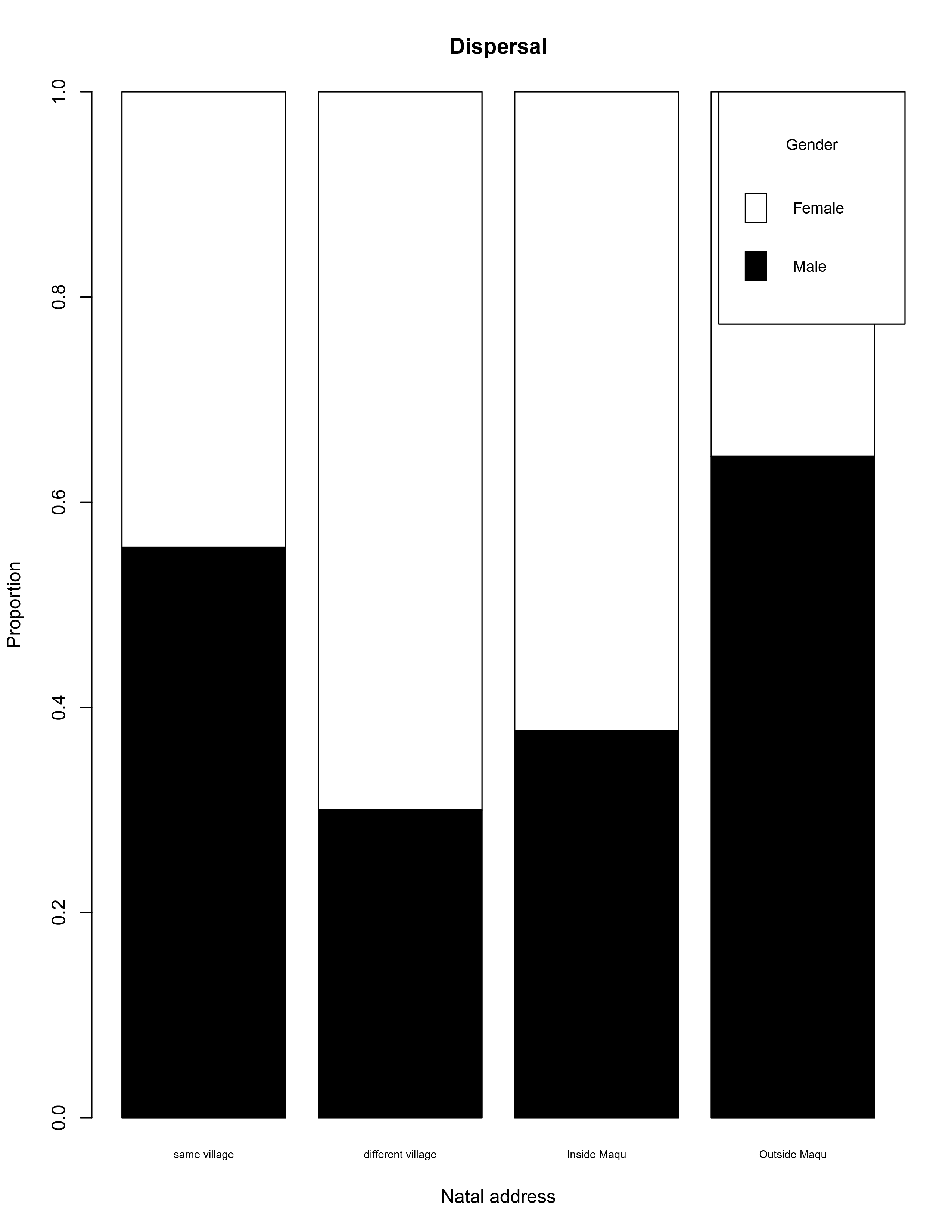


**SI Figure1**: **dispersal information of males and females**. Four different categories of home address are included in the data site. The difference in the natal address as a function of sex is significant, χ² (3, N =1320) = 60.703, p < 0.001

622 male and 698 females are included in the database. We asked each individual about the address of their natal house and divided the address into 4 categories. If their natal house is the same as their current house then they are in the “same village” (male= 356 female=284 ), and in each township, there are several villages or “sheng chan dui’; each village/”sheng chan dui“ within the same township are geographically close to each other and governed by the same village leader. Maqu has 7 townships, and in this data, there are 42 males and 98 females who are living in the same township as their natal house but in different villages/”sheng chan dui”. There are some individuals (male=175, female= 289) who were born in other townships a bit further away from their current house. There are also a small number of individuals from outside of Maqu county, and there are significantly more males than females from outside of the county. The difference in the address of the natal house as a function of sex is significant, with females being more likely to have dispersed between villages, χ²(3, N =1320) = 60.703, p < 0.001

**Marital status:**

Monogamy is the principle form of marriage (in line with current Chinese law). Polygamous marriage is much less frequent than in recent history; there are 5 men and 10 women who described themselves as in polygynous relationships, and 5 men and 5 women who are in polyandrous relationships among those we interviewed. We then divided marital status into two types: single and married based on their current marital status. Females are significantly more likely to stay single than males (although some may have been in unreported polygamous relationships given that polygamous marriage is not recognised in Chinese law)( RR=0.34, 95% CI= (0.14, 0.80), P <0.001).


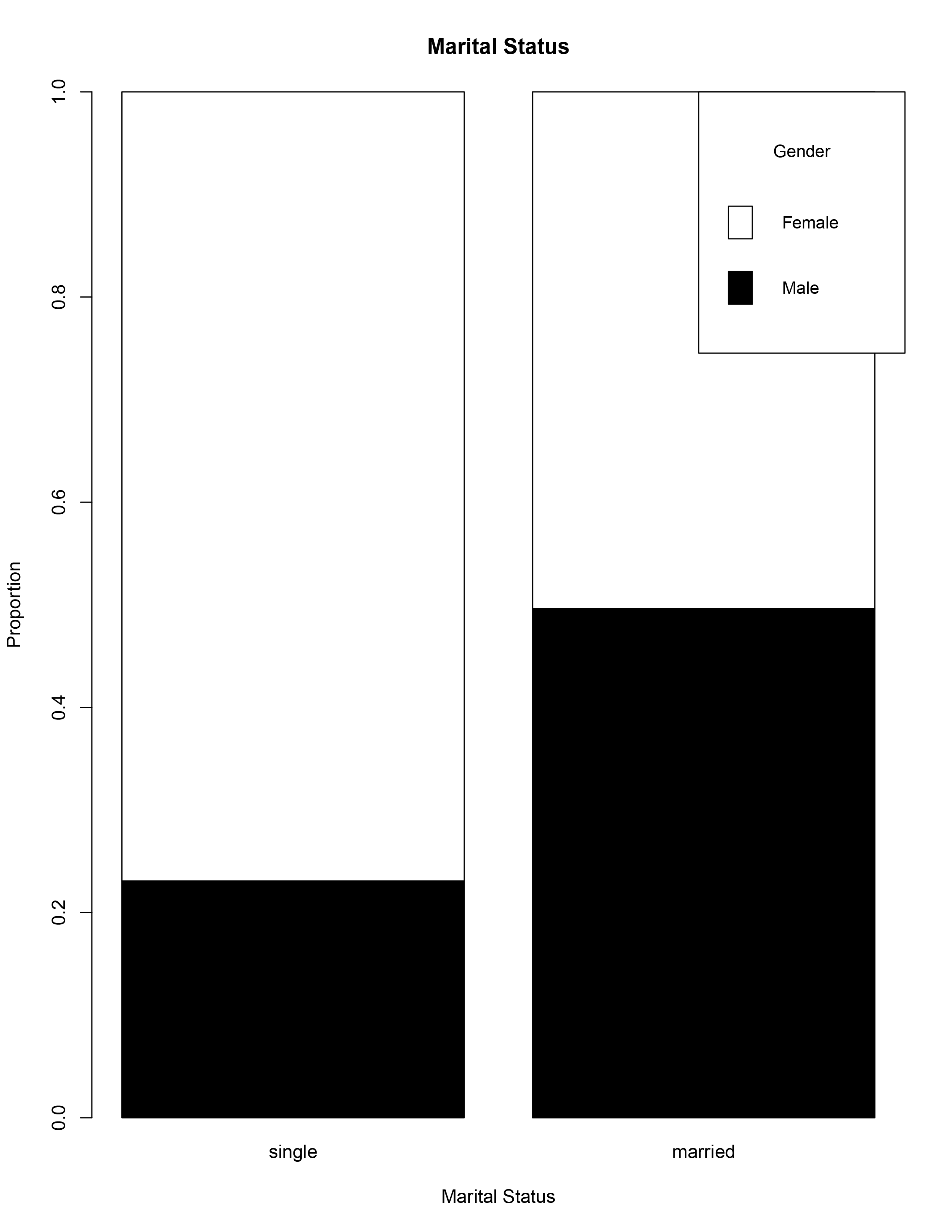


**SI Figure2: Bar plot of the Marital status of males and females** who ever had children. The black bar represents adult males; white bar represents adult females.

**Self-reported gender preferences:**


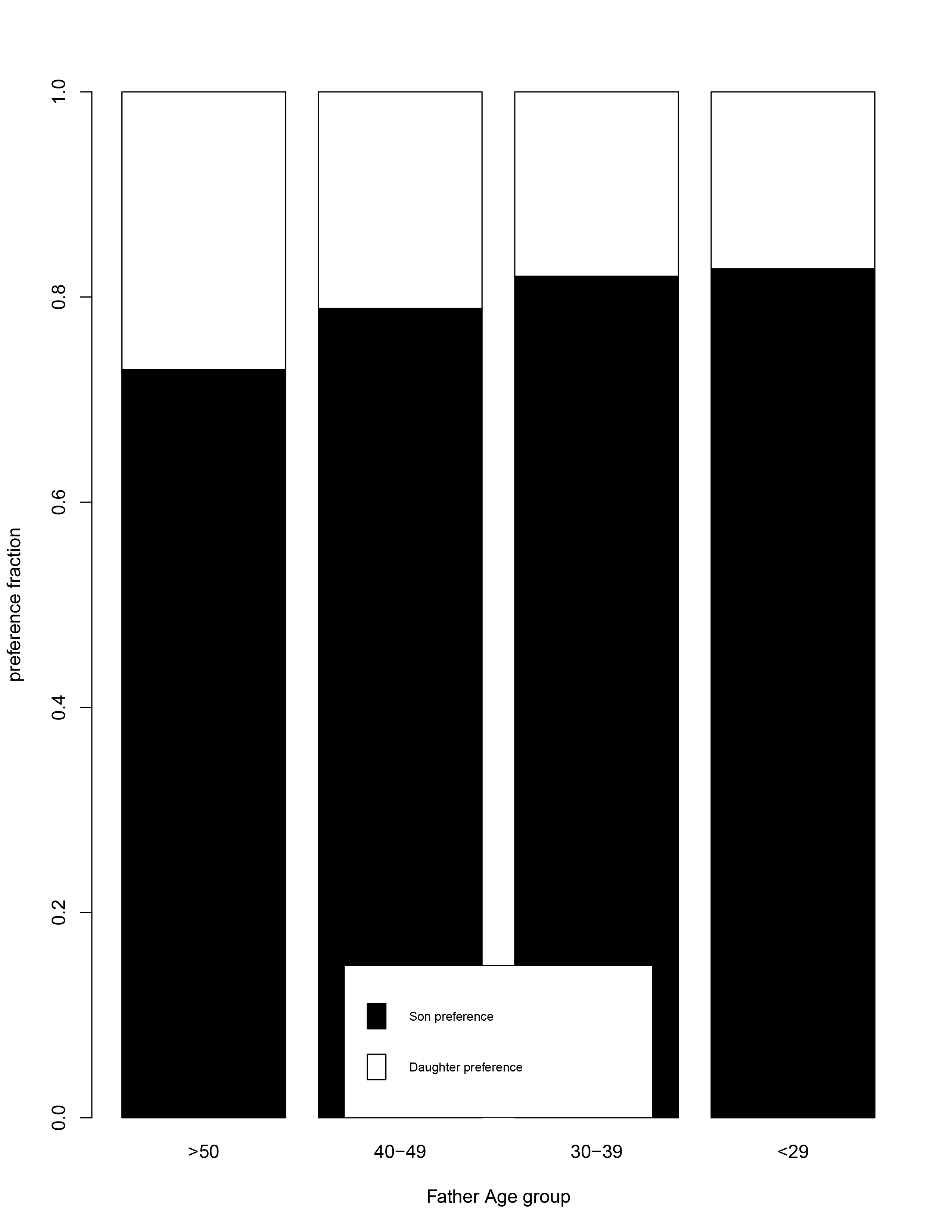


**SI Figure3: male self-reported offspring sex preference in 4 different age groups.** white bars indicate a preference for a daughter, the black bars indicate a preference for a son.


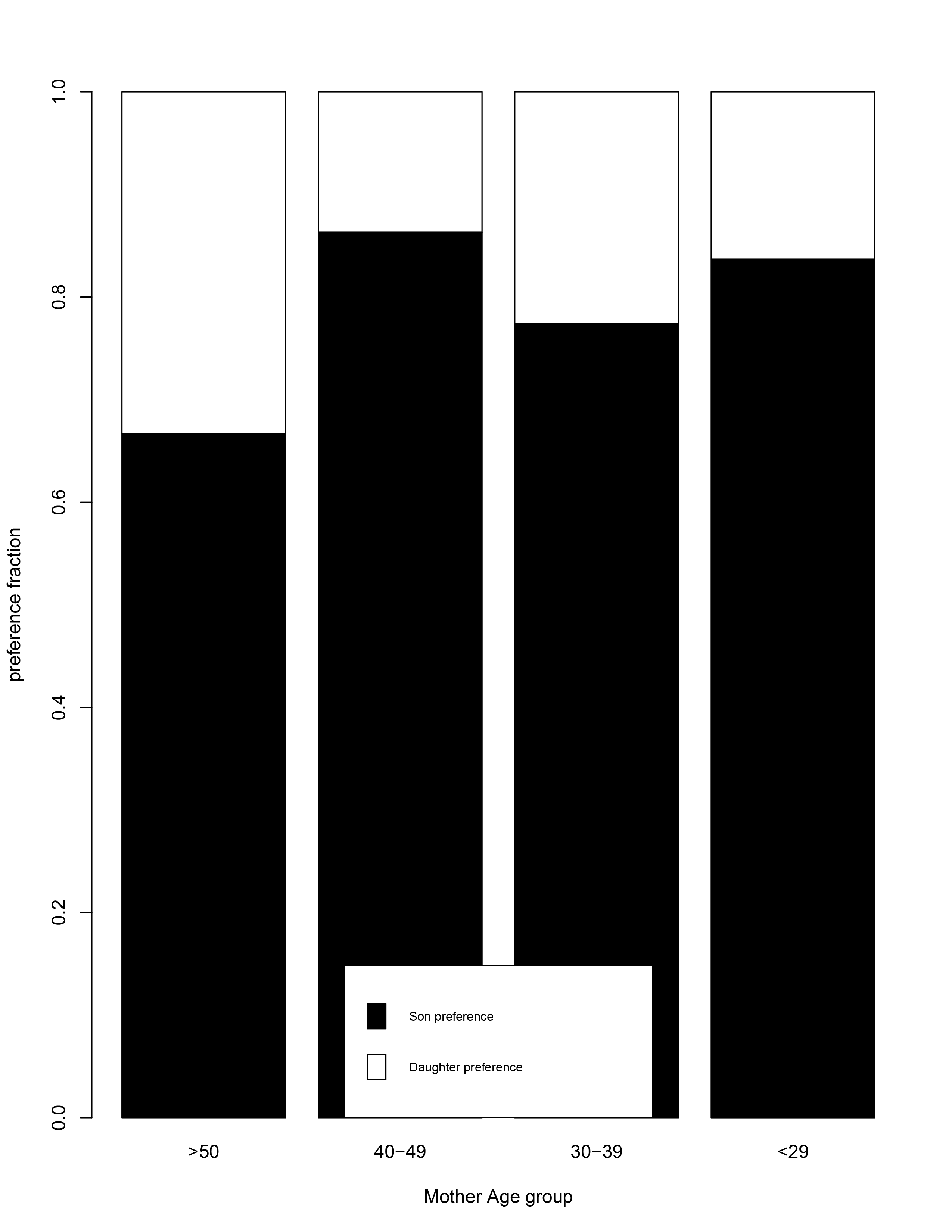


**SI Figure4: female self-reported offspring sex preference in 4 different age groups.** white bar indicates a preference for a daughter, the black bar indicates a preference for a son.

| **Variables** | **OR (95% CI)** | **P value** |
| --- | --- | --- |
| Female reporter (ref: male reporter) | 0.90 (0.51,1.61) | 0.730 |
| Age group (ref: <29) |  |  |
| 30-39 | 1.27(0.72,2.21) | 0.407 |
| 40-49 | 1.07(0.60,1.90) | 0.831 |
| **>50** | **2.27(1.29,3.98)** | **0.004**** |
| **Yak** | **0.99(0.99,1.00)** | **0.039*** |
| Yak*Female reporter | 1.00(0.99, 1.01) | 0.733 |

*p<0.05, **p<0.01, ***p<0.001

**SI Table 1: Logistic regression of self-report preference from adult males and females on preference for daughters.** OR indicates odds ratio, 95% CI means 95% confidence intervals. Statistical significance indicates in bold.

**SI Table 2: Table of sample sizes for:** Child mortality; Birth interval; Breastfeeding and Bottle-feeding. Showing the number of mothers and the number of children of each sex.

| **Candidate models** | **Loglik** | **AICc** | **delta** | | **weight** |
| --- | --- | --- | --- | --- | --- |
| **1. Control+cohort +yak+ yak*gender+sexorder** | **-2687.87** | **5773.105** | | **0.000** | **0.862** |
| 2. Control+**cohort*gender** +yak+yak*gender+sexorder | -2688.184 | 5776.804 | 3.699 | | 0.135 |
| 3. Control | -2709.113 | 5784.918 | 11.813 | | 0.002 |

**SI Table 3**: Model selection for the mortality analysis before age 5. Candidate models were ranked on the ascending order based on the number of AICc.

| **Candidate models** | **Loglik** | **AICc** | **Delta** | **weight** |
| --- | --- | --- | --- | --- |
| 1. Control + cohort   + yak + yak*gender+ sex order | -5829.901 | 11677.95 | 0.000 | 0.934 |
| 2. Control + **cohort*gender** +yak+yak*gender+ sex order | -5828.475 | 11683.26 | 5.305 | 0.066 |
| 3. Control | -5900.822 | 11805.65 | 127.700 | 0.000 |

**SI Table 4**: Model selection for the interbirth intervals analysis. Candidate models were ranked in ascending order based on the AICc.

| **IBI** | | | | | | | | |
| --- | --- | --- | --- | --- | --- | --- | --- | --- |
| **cohort** | min | | max | | mean | | sd | |
| **<1980** | 0.00 | | 5.00 | | 2.78 | | 1.33 | |
| **1981-1989** | 0.00 | | 5.00 | | 2.38 | | 1.27 | |
| **1990-2000** | 0.00 | | 5.00 | | 2.4 | | 1.35 | |
| **>2001** | 0.00 | | 5.00 | | 2.01 | | 1.12 | |
| **overall** | 0.00 | | 5.00 | | 2.32 | | 1.28 | |
| **Number of yaks** | | | | | | | | |
| **cohort** | min | | max | | mean | | sd | |
| **<1980** | 0.00 | | 205.00 | | 63.75 | | 42.13 | |
| **1981-1989** | 0.00 | | 300.00 | | 70.79 | | 50.21 | |
| **1990-2000** | 0.00 | | 300.00 | | 65.90 | | 49.63 | |
| **>2001** | 0.00 | | 270.00 | | 54.06 | | 39.92 | |
| **overall** | 0.00 | | 300.00 | | 62.14 | | 45.96 | |
| **Child death before age 5** | | | | | | | | |
|  | Son | |  | | Daughter | | | |
|  | Death | | living | | Death | | Living | |
| **<1980** | 33 | | 193 | | 16 | | 193 | |
| **1981-1989** | 71 | | 242 | | 35 | | 227 | |
| **1990-2000** | 69 | | 366 | | 62 | | 330 | |
| **>2001** | 78 | | 454 | | 53 | | 426 | |
| **overall** | 251 | | 1255 | | 165 | | 1176 | |
| **Bottle feeding before 12 months** | | | | | | | | |
|  | Son | |  | | Daughter | | | |
|  | Start bottle | Not start | | | | Start bottle | | Not start |
| **1990-2000** | 16 | 20 | | | | 8 | | 24 |
| **2001-2010** | 77 | 48 | | | | 56 | | 47 |
| **>2010** | 38 | 21 | | | | 29 | | 26 |
| **overall** | 131 | 89 | | | | 93 | | 97 |
| **Breast feeding before 12 months** | | | | | | | | |
|  | Son | |  | | Daughter | | | |
|  | Not stop | | Stop | Not stop | | | | Stop |
| **1990-2000** | 25 | | 11 | 30 | | | | 2 |
| **2001-2010** | 87 | | 38 | 79 | | | | 24 |
| **>2010** | 42 | | 17 | 44 | | | | 11 |
| **overall** | 154 | | 66 | 153 | | | | 37 |

**SI Table 5**: descriptive statistics about the length of the Interbirth intervals in years (IBI), number of yaks and events of child death before age 5. (0 means less than 1 year; birth intervals were censored at 5 years)
